# Advances in Development of Drug Treatment for Hemophilia with Inhibitors

**DOI:** 10.1021/acsptsci.4c00560

**Published:** 2024-11-08

**Authors:** Surasak Wichaiyo

**Affiliations:** † Department of Pharmacology, Faculty of Pharmacy, Mahidol University, Bangkok, 10400 Thailand; ‡ Centre of Biopharmaceutical Science for Healthy Ageing, Faculty of Pharmacy, Mahidol University, Bangkok, 10400 Thailand

**Keywords:** hemophilia with inhibitors, bypassing agents, bispecific antibody, anti-TFPI antibody, antithrombin siRNA

## Abstract

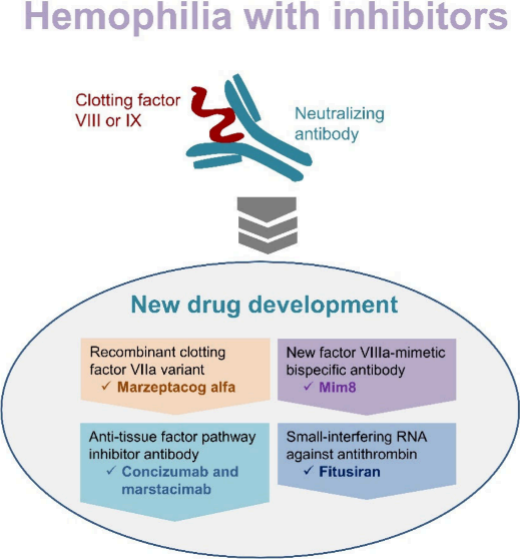

Patients with hemophilia A and B who have inhibitors face limited treatment options, because replacement therapy with clotting factor VIII or IX concentrates is ineffective, particularly for patients with high-titer inhibitors. Current mainstay therapies include immune tolerance induction (through frequent injections of clotting factor VIII or IX concentrates) to eradicate inhibitors and bypassing agents (such as recombinant activated clotting factor VII and activated prothrombin complex concentrates) for the prevention and treatment of bleeding episodes. The use of these agents typically requires intravenous injections and sometimes hospitalization, which can be burdensome for patients. More recently, emicizumab, a bispecific antibody that mimics the function of activated clotting factor VIII, has demonstrated favorable efficacy for prophylaxis in patients with hemophilia A and inhibitors, representing a promising new therapeutic strategy. Ongoing research aims to discover and develop easy-to-use nonfactor agents for managing hemophilia with inhibitors. This review summarizes the current understanding of the pathophysiology of inhibitor development in hemophilia, outlines existing treatment options, and discusses advancements in novel therapeutic biologics, including a recombinant activated clotting factor VII variant (marzeptacog alfa), a new bispecific antibody (Mim8), antitissue factor pathway inhibitor antibodies (concizumab and marstacimab), and small interfering RNA targeting antithrombin (fitusiran). All of these agents are administered subcutaneously, with some offering the convenience of less frequent dosing (e.g., weekly or monthly). These potential drug candidates may provide significant benefits for the prophylaxis or treatment of bleeding disorders in patients with hemophilia and inhibitors.

Hemophilia A and hemophilia B result from mutations in the genes that encode clotting factor VIII (FVIII) and clotting factor IX (FIX), respectively.^1,2^ Most patients with hemophilia are male because of the condition’s X-linked recessive inheritance pattern.^1,2^ FVIII or FIX deficiency disrupts the contact activation (intrinsic) pathway of coagulation, leading to a hemostatic disorder.^1,2^ Patients with severe hemophilia A or B require regular replacement therapy with FVIII or FIX, respectively, to maintain factor levels at 1%–3% of the normal value to prevent bleeding diathesis (e.g., hemarthrosis) and its complications (e.g., arthrosis and arthropathy).^3^ Many physicians prefer target factor levels of 3%–5% of the normal value to achieve optimal treatment outcomes.^3^

Currently available FVIII and FIX concentrates are produced either from human plasma or via recombinant DNA technology using human or nonhuman cells. In addition, protein modification techniques have been employed to extend the plasma half-life of recombinant clotting factors, reducing the frequency of intravenous injections required (Table 1). However, because of the nonself-origin of both plasma-derived and recombinant FVIII and FIX concentrates, patients with hemophilia who receive these products may develop neutralizing antibodies against FVIII or FIX, a condition known as “hemophilia with inhibitors.”^4,5^ The incidence of neutralizing antibody production is approximately 20%–30% in patients with severe hemophilia A (FVIII levels of <1% of the normal value),^4,6,7^ while lower rates are observed in patients with moderate hemophilia A and severe hemophilia B (5%–10%).^4,6,7^

**Table 1:** Characteristics of FVIII and FIX Concentrates^a^

Clotting factor concentrates	Source	Half-life (hours)
Plasma-derived FVIII^8^	Pooled human plasma	12–18
rFVIII (-octocog)^8^ (e.g., rFVIII formulated with sucrose, moroctocog alfa, turoctocog alfa, simoctocog alfa)	Recombinant DNA technology using cell lines (HEK, CHO, BHK)	5–15
**Extended half-life rFVIII**(9,10)		
Efmoroctocog alfa	HEK, Fc-fusion	19
Rurioctocog alfa pegol	CHO, PEGylation	14–16
Turoctocog alfa pegol	CHO, PEGylation	19
Damoctocog alfa pegol	BHK, PEGylation	19
Lonoctocog alfa	CHO, single-chain rFVIII with covalent link between heavy and light chains	14.5
**Sustained half-life rFVIII**(11)		
Efanesoctocog alfa	HEK, B domain-deleted single-chain rFVIII connected to D’D3 domain of von Willebrand factor	37–42
Plasma-derived FIX^12^	Pooled human plasma	29–43
rFIX (-nonacog) (e.g., nonacog alfa)	Recombinant DNA technology using cell lines (HEK, CHO, BHK)	18–24^12^ (new data have revealed a longer half-life of 36–50 h^13^)
**Extended half-life rFIX**(9,10)		
Eftrenonacog alfa	HEK, Fc-fusion	82
Albutrepenonacog alfa	CHO, albumin fusion	102
Nonacog beta pegol	CHO, PEGylation	93

a HEK = human embryonic kidney cells, CHO = Chinese hamster ovary cells, BHK = baby hamster kidney cells, rFVIII = recombinant clotting factor VIII, rFIX = recombinant clotting factor IX.

Because FVIII or FIX replacement therapy is not effective in patients with hemophilia with inhibitors, alternative treatment options play a central role in preventing and managing bleeding disorders in these individuals.^4,5^ This review provides a brief overview of the current understanding of the pathophysiology and treatment of hemophilia with inhibitors, discusses new therapeutic agents, and evaluates their pharmacological properties, benefits, and associated challenges.

## 2 Pathophysiology of Inhibitor Development in Hemophilia

**Figure 1:**
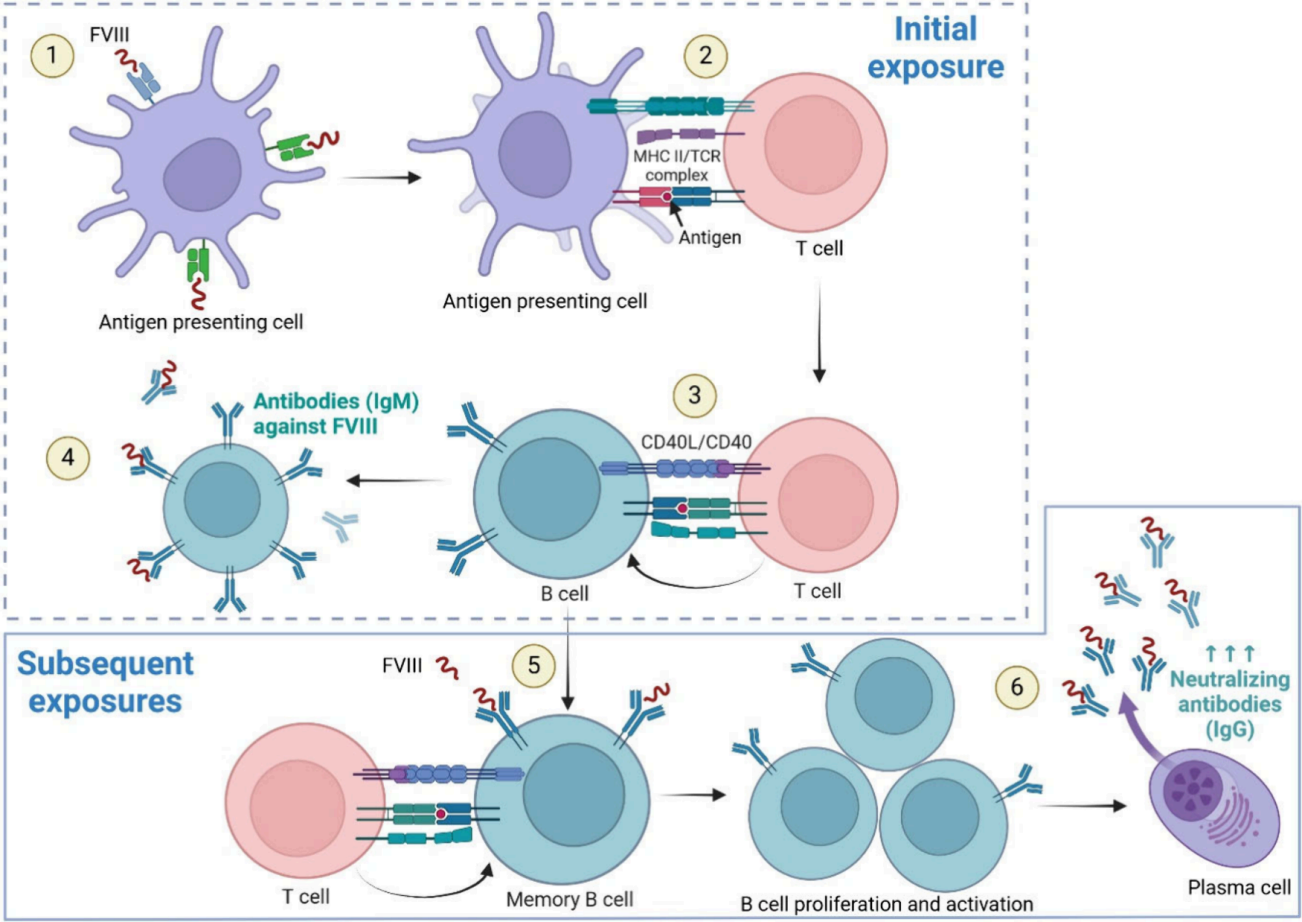
Proposed mechanism of neutralizing antibody production in hemophilia with inhibitors. At the initial exposure to FVIII or FIX concentrates, these biologics may be recognized as foreign antigens. (1) Antigen-presenting cells recognize and engulf the antigen and then (2) present it to T cells via a MHC II–TCR interaction. (3) Subsequently, the activated T cells stimulate B cells, leading to (4) the initial release of IgM against FVIII or FIX and (5) the generation of memory B cells. Upon subsequent exposures, memory B cells act as antigen-presenting cells, (5) stimulating T cells to further induce B-cell proliferation, activation, and differentiation into plasma cells, which (6) produce greater amounts of neutralizing IgG antibodies. (Created in BioRender. Wichaiyo, S. (2024) BioRender.com/z49s578.) FVIII = clotting factor VIII, FIX = clotting factor IX, MHC II = major histocompatibility complex class II, TCR = T cell receptor, IgM = immunoglobulin M, IgG = immunoglobulin G.

The mechanism of neutralizing antibody production following exposure to FVIII and FIX concentrates has been proposed (Figure 1).^5,14,15^ Upon the initial use of clotting factor concentrates, antigen-presenting cells (APCs) may recognize FVIII or FIX as antigens and engulf them. The APCs then present the antigen to T cells via a major histocompatibility complex class II–T cell receptor interaction, stimulating an immune response. The activated T cells interact with B cells, promoting the initial release of immunoglobulin M (IgM) against FVIII or FIX and generating memory B cells that recognize these clotting factors (Figure 1). During subsequent exposures to clotting factor concentrates, memory B cells act as APCs, which trigger T cells to induce B-cell proliferation and activation (Figure 1). The activated B cells then differentiate into plasma cells to produce greater amounts of FVIII or FIX neutralizing immunoglobulin G (IgG) antibodies, known as inhibitors.^5,14,15^ Detectable levels of inhibitors typically appear after 10–75 days of exposure to FVIII and FIX concentrates.^5,14,16^ Consequently, frequent monitoring for inhibitor development in patients with hemophilia during the initial uses of FVIII and FIX concentrates is recommended.^4,5^ Hemophilia with inhibitors might not present clinical manifestations different from those seen in patients without inhibitors, but failure of prophylaxis and acute bleeding treatment are commonly observed at standard doses of clotting factor concentrates.^17^

The inhibitor titer can be detected in plasma using the Bethesda assay or the Nijmegen modification of the Bethesda assay and is reported as Bethesda units per milliliter (BU/mL).^4,5,18,19^ 1 BU/mL is defined as the dilution of patient plasma required to neutralize 50% of the FVIII or FIX activity in an equivalent volume of normal plasma.^18^ Patients are considered to have developed an inhibitor if the antibody titer exceeds 0.6 BU/mL in two consecutive tests conducted 1–4 weeks apart. They are classified as having low-titer inhibitors (low-responding inhibitors) if the antibody titer is <5 BU/mL and as having high-titer inhibitors (high-responding inhibitors) if the antibody titer is ≥5 BU/mL.^4,5,18,19^ Patients with low-titer inhibitors, including those with transient inhibitors that resolve within 6 months, may receive increased doses of clotting factor concentrates.^4,18^ By contrast, high-titer inhibitors require alternative therapeutic strategies.^4,18^

## 3 Current Treatment for Hemophilia with Inhibitors

Management of hemophilia with high-titer inhibitors involves three main strategies: eradication of the inhibitor through immune tolerance induction (ITI), prophylaxis with bypassing agents or an FVIIIa-mimetic bispecific antibody, and treatment of bleeding episodes with bypassing agents.^4,5,20^

### 3.1 ITI

For patients with high-titer inhibitors, particularly those with hemophilia A, it is recommended to initiate ITI (repeated and frequent administrations of clotting factor concentrate) as early as possible.^4,20^ ITI is the standard of care for inhibitor eradication.^15,20^ It has been suggested that ITI may attenuate immune responses by inhibiting memory B-cell differentiation or inducing T-cell anergy.^15,21^ Common ITI protocols include the high-dose FVIII regimen (Bonn protocol), low-dose FVIII regimen (Van Creveld protocol), or high-dose FVIII combined with immunosuppressants (e.g., cyclophosphamide and IgG) and immunoadsorption (Malmö protocol) in patients with hemophilia A and inhibitors.^15,20^ ITI has a success rate of approximately 70%–80%,^15,20^ although relapse may occur in 12%–15% of patients.^22,23^ Because ITI requires several months to complete, it imposes a significant burden on patients, including issues related to venous access.^4,5,15,24^ Importantly, ITI is generally not recommended for patients with hemophilia B and inhibitors because of its low success rate and the potential risks, such as allergic reactions and the development of nephrotic syndrome.^20,25^

### 3.2 Prophylaxis

**Figure 2:**
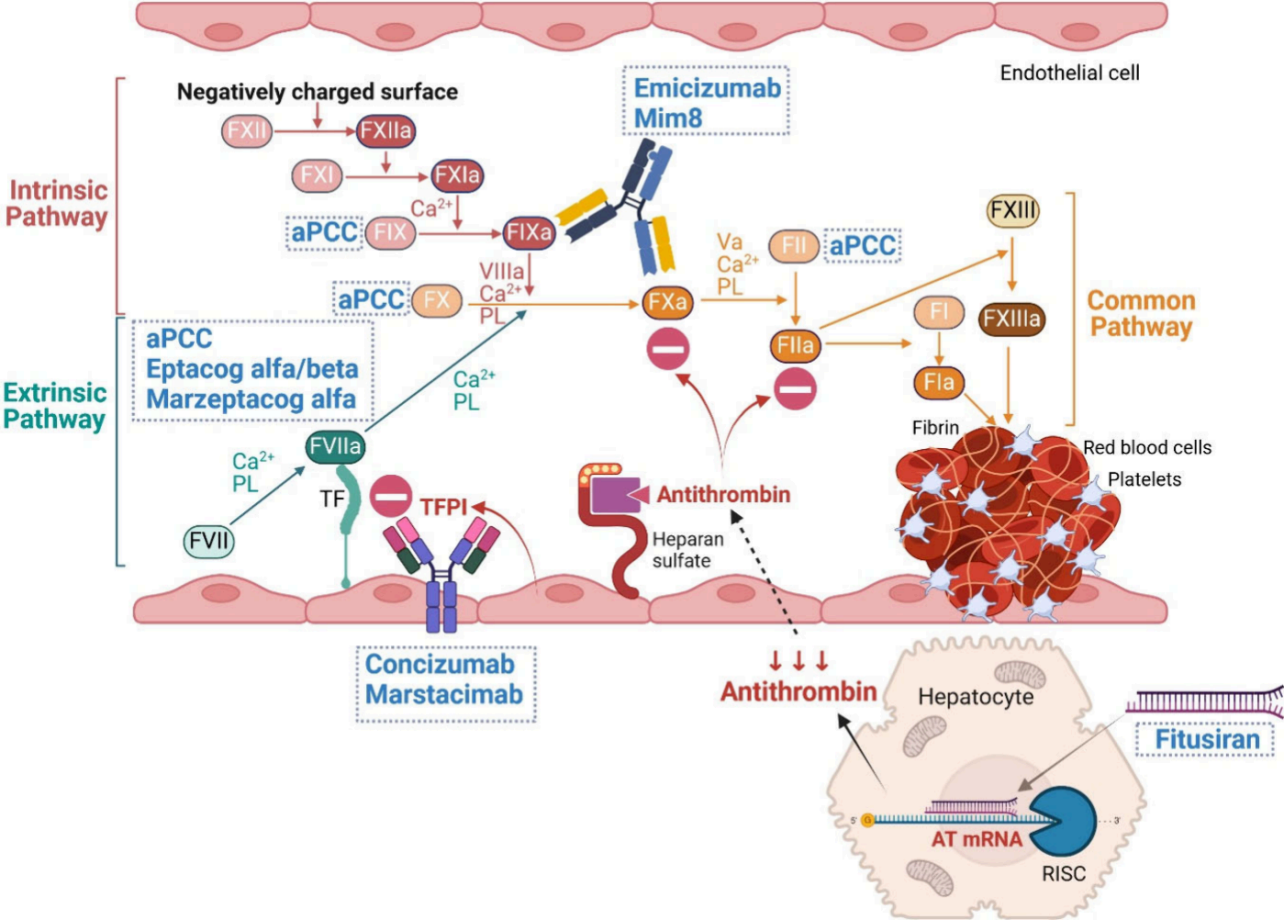
Sites of action of current and potential therapeutic agents for hemophilia with inhibitors. **Eptacog alfa/beta** and **marzeptacog alfa** are rFVIIa and an rFVIIa variant, respectively, which function as bypassing agents by binding to TF to activate the extrinsic pathway of coagulation. **aPCC** contains FVIIa, FIX, FIXa, FX, FXa, FII, and FIIa and acts as a bypassing agent that directly activates both extrinsic and common pathways. **Emicizumab** and **Mim8** are bispecific antibodies; one Fab region of these antibodies binds to FIXa, while the other Fab binds to FX, mimicking the function of FVIIIa in the activation of FXa through the intrinsic pathway. **Concizumab** and **marstacimab** are anti-TFPI antibodies. By inhibiting TFPI, these antibodies enhance the activity of the TF–FVIIa complex, promoting clot formation. **Fitusiran** is an siRNA that induces RISC to specifically degrade AT mRNA in hepatocytes. A reduction in AT synthesis leads to increased activity of FXa and thrombin, facilitating fibrin clot formation. (Created in BioRender. Wichaiyo, S. (2024) BioRender.com/c75q143.) rFVIIa = recombinant activated clotting factor VII, TF = tissue factor, aPCC = activated prothrombin complex concentrate, FIIa = thrombin, Fab = fragment antigen-binding, FVIIIa = activated clotting factor VIII, FXa = activated clotting factor X, TFPI = tissue factor pathway inhibitor, siRNA = small interfering RNA, RISC = RNA-induced silencing complex, AT = antithrombin.

FVIII and FIX play key roles in forming the tenase complex, which generates activated clotting factor X (FXa) and thrombin via the contact activation (intrinsic) pathway (Figure 2). Because hemophilia with high-titer inhibitors does not respond to clotting factor replacement therapy, prophylaxis in these patients requires agents that act through alternative (bypassing) pathways, involving clotting factors from the tissue factor (extrinsic) and/or common pathways. Currently available bypassing agents include recombinant activated clotting factor VII (rFVIIa) and activated prothrombin complex concentrate (aPCC), both of which have been in clinical use for >20 years.^5,20^ The FVIIIa-mimetic bispecific antibody emicizumab was recently approved for prophylaxis in patients with hemophilia A, with or without inhibitors.^26,27^

rFVIIa contributes to the tenase complex (together with tissue factor) in the extrinsic pathway (Figure 2), enabling it to act as a bypassing clotting factor that drives hemostasis in patients with hemophilia and inhibitors. Available rFVIIa products include eptacog alfa (NovoSeven), which is produced in baby hamster kidney cells, and eptacog beta (SEVENFACT and CEVENFACTA), derived from the milk of FVII transgenic rabbits.^29,30^ Eptacog alfa may be used for secondary prophylaxis of bleeding in patients with hemophilia A or B, particularly those with high-titer inhibitors and a history of previous bleeds.^29−32^ In a phase 2 clinical study, the prophylactic dose of eptacog alfa was reported to be 90 μg/kg or 270 μg/kg administered via intravenous injection daily, while phase 4 postmarketing surveillance studies showed varying doses.^31,32^ These trials indicated a reduction in bleeding frequency by 45%–60% without any reported thrombotic events.^29−33^ Eptacog beta, however, is not indicated for prophylaxis.^29,30^

aPCC (FEIBA, factor eight inhibitor bypassing activity) contains plasma-derived vitamin K-dependent clotting factors, primarily FVIIa, FX, FIX, and prothrombin, with minor amounts of FXa, FIXa, and thrombin (Figure 2).^33,34^ aPCC requires less frequent dosing but has a larger infusion volume than rFVIIa. It is indicated for both prophylaxis and acute treatment of bleeding in patients with hemophilia A or B with inhibitors. The recommended dose for routine prophylaxis is 85 units/kg every other day (three times a week).^4,33,35^ A clinical study in patients with hemophilia and inhibitors (Pro-FEIBA trial) reported that aPCC reduced overall bleeding episodes by approximately 60%, with a 72% reduction in target-joint bleeding (defined as ≥3 hemarthroses in a single joint during the treatment period).^36^ No thrombotic events were reported during prophylaxis with aPCC.^36^

Emicizumab is a monoclonal antibody that simultaneously binds to FIXa and FX. This bispecific antibody acts as a functional mimic of FVIIIa and is often referred to as an “FVIIIa mimetic,” although it has no structural homology to FVIIIa. One fragment antigen-binding (Fab) region of emicizumab binds to FIXa, while the other Fab binds to FX on the plasma membrane of activated platelets, promoting FIXa-driven activation of FX (Figure 2).^26,27^ Mechanistically, emicizumab benefits patients with hemophilia A, independent of inhibitor development.^26,27^ Clinical studies have consistently shown that emicizumab is effective for prophylaxis, achieving an 80%–90% success rate in reducing bleeding episodes in patients with hemophilia A with or without inhibitors.^26,27^ Consequently, emicizumab is currently approved for prophylaxis in patients with hemophilia A, regardless of their inhibitor status.^26,27^

Emicizumab is administered subcutaneously, offering 80%–90% bioavailability with a plasma half-life of approximately 4–5 weeks.^26^ The recommended loading dose is 3 mg/kg as a subcutaneous injection once weekly for 4 weeks, followed by a maintenance dose of either 1.5 mg/kg once weekly, 3 mg/kg every 2 weeks, or 6 mg/kg every 4 weeks.^26,27^ Emicizumab is now considered a first-line drug for prophylaxis in patients who have hemophilia A with inhibitors, although ITI remains the standard therapy.^24^ Additionally, emicizumab may be a valuable alternative for patients who face challenges with ITI because those on emicizumab prophylaxis require less frequent dosing of FVIII during ITI.^24^

Emicizumab generally has a favorable safety profile, with injection site reactions (approximately 26%) being the most common side effect. However, a few thrombotic events have been reported, particularly when emicizumab is used concurrently with high doses of aPCC.^26,27^ For instance, in the HAVEN 1 study, which investigated emicizumab prophylaxis in patients with hemophilia A with inhibitors, two cases of thrombotic microangiopathy and two cases of thrombosis were reported.^37^ These incidents occurred in patients who had received multiple doses of aPCC for breakthrough bleeding.^37^ Among 37 cases of non-aPCC-related thromboembolic events following emicizumab prophylaxis, it was found that most cases (92%) had underlying cardiovascular or thrombotic risk factors.^26,27^

Notably, emicizumab can interfere with certain laboratory tests, such as the activated partial thromboplastin time, but not the prothrombin time.^38^ Chromogenic assays for FVIII levels and FVIII inhibitors should be performed using bovine-derived FIXa and FX components because emicizumab affects test results when human-derived FIXa and FX components are used.^38^ Antidrug antibodies (approximately 5%) have been observed in clinical studies of emicizumab.^26,27^ Measuring the plasma emicizumab level is recommended in patients suspected to have inhibitor development against emicizumab.^38^

### 3.3 Treatment of Bleeding Episodes

An increased dose of FVIII or FIX concentrates can be used to treat bleeding episodes in patients with low-titer inhibitors.^4,20^ For patients with high-titer inhibitors, bypassing agents are the primary treatment option. The recommended dose of eptacog alfa (NovoSeven) for managing acute bleeding and perioperative care is 90–120 μg/kg, administered as an intravenous injection every 2–3 h until hemostasis is achieved, with a plasma half-life of 2–3 h for rFVIIa.^4,29,30^ This treatment has a reported success rate of 80%–90%.^29,30,33^ Eptacog beta (SEVENFACT and CEVENFACTA) is also indicated for treating bleeding episodes in patients with hemophilia A or B with inhibitors.^29,30^ In cases of mild to moderate bleeding, eptacog beta may be administered as a 75-μg/kg intravenous injection every 3 h until hemostasis or as a 225-μg/kg loading dose, followed by 75 μg/kg every 3 h if hemostasis is not achieved within 9 h.^29,30^ For severe bleeding, a loading dose of 225 μg/kg of eptacog beta is recommended, followed by 75 μg/kg every 2 h if hemostasis is not achieved within 6 h.^29,30^ The success rate of eptacog beta is comparable to that of eptacog alfa.^29,30^ Thrombotic events following the use of rFVIIa are relatively rare.^29,30,33^ Most patients who experience thrombotic events during rFVIIa treatment have predisposing thrombotic risk factors.^39^ rFVIIa is the preferred option for treating breakthrough bleeding in patients receiving emicizumab prophylaxis.^39^

For aPCC (FEIBA), prothrombin and FX are the primary contributors to the hemostatic effect, with FVIIa playing a complementary role.^40^ The usual dose of aPCC is 50–100 units/kg administered via intravenous infusion every 8–12 h until hemostasis is achieved, and it is indicated for treating bleeding and for perioperative management in patients who have hemophilia with inhibitors. The success rate of aPCC is approximately 80%, which is comparable to that of rFVIIa.^4,33,35^ Adverse thrombotic events associated with aPCC are very rare.^35,40^ In patients with a history of anaphylaxis to FIX-containing products, particularly those with hemophilia B and inhibitors, rFVIIa is preferable to aPCC.^4,33,35^ Moreover, the use of aPCC to treat bleeding should be avoided in patients receiving emicizumab prophylaxis because it may increase the risk of thrombotic events.^39,40^ If aPCC is necessary, it should be used for the short-term (no longer than 24 h) at an initial dose of ≤50 units/kg, and the total dose should not exceed 100 units/kg/day.^39^

## 4 Novel Potential Therapeutic Agents

The development of new therapeutic modalities for prophylaxis and treatment of bleeding disorders in patients who have hemophilia with inhibitors is ongoing. These modalities include next-generation recombinant clotting factors and nonfactor approaches (Table 2).

**Table 2:** New Therapeutic Approaches for Hemophilia with Inhibitors^a^

Name	Target	Route	Potential benefits	Status
Marzeptacog alfa (activated)	rFVIIa variant with substitutions of four amino acids to increase activity and prolong plasma half-life	s.c. daily	Phase 2 study:^41^ Prophylactic dose escalation (30–120 μg/kg) of marzeptacog alfa (activated) showed >90% reduction in bleeding episodes in hemophilia with inhibitors.	Phase 3 study (on-demand treatment of bleeding episodes): terminated (Crimson 1 study, NCT04489537)
				Phase 3 study (prophylaxis): unknown
Mim8 (denecimig)	FVIIIa-mimetic bispecific antibody	s.c. weekly or monthly	Phase 2 study:^42^ Prophylaxis with Mim8 resulted in 70%–90% of zero bleeds in hemophilia A.	Phase 3 studies (prophylaxis): ongoing (FRONTIER2 and FRONTIER3 trials)
Concizumab (mAb 2021)	Anti-TFPI antibody	s.c. daily	Phase 3 study:^43^ Prophylaxis with loading dose of 1.0 mg/kg concizumab, followed by 0.2 mg/kg reduced bleeding episodes in hemophilia with inhibitors.	Approved in Canada: prophylaxis in hemophilia B patients with inhibitors
				US FDA: requested additional information for monitoring and dosing
Marstacimab	Anti-TFPI antibody	s.c. weekly	Phase 1*b*/2 study:^44^ Prophylaxis with marstacimab significantly reduced bleeding rate in hemophilia with or without inhibitors.	Phase 3 study in patients with severe hemophilia A or moderately severe to severe hemophilia B (BASIS trial, NCT03938792): Recently reported^45^
				US FDA: approved in October 2024 for prophylaxis in hemophilia A or B without inhibitors
Fitusiran	AT siRNA	s.c. monthly	Phase 3 study:^46−48^ Prophylaxis with fitusiran 80 mg significantly reduced bleeding frequency in hemophilia with or without inhibitors.	US FDA: under review

a rFVIIa = recombinant activated clotting factor VII, FVIIIa = activated clotting factor VIII, TFPI = tissue factor pathway inhibitor, AT siRNA = antithrombin small interfering RNA, s.c. = subcutaneous injection, US FDA = United States Food and Drug Administration.

### 4.1 rFVIIa Variant

A new rFVIIa variant, marzeptacog alfa (activated), is currently under investigation (Figure 2). Marzeptacog alfa (activated) was developed by substituting two amino acids in the catalytic domain of rFVIIa to enhance its activity, along with two additional amino acid modifications at the N-terminus for glycosylation, which helps prolong its plasma half-life.^49^ Despite these modifications, marzeptacog alfa (activated) exhibits only a slightly longer half-life (3.0–3.5 h) than wild-type rFVIIa following intravenous injection.^49^ Notably, marzeptacog alfa (activated) is resistant to protease degradation, which potentially enables its use as a subcutaneous injection.^41^ Although subcutaneous marzeptacog alfa (activated) has a longer half-life (17 h), its bioavailability is relatively low (27%), necessitating higher doses than the intravenous route.^41^

A phase 2 clinical study in patients with hemophilia and inhibitors reported that prophylaxis with marzeptacog alfa (activated), administered as a subcutaneous dose-escalation injection (30–120 μg/kg) daily for 50 days, resulted in a > 90% reduction in bleeding episodes.^41^ Injection site reactions were the most common side effects observed.^41^ Currently, there are no phase 3 studies reporting the efficacy and safety of marzeptacog alfa (activated) for prophylaxis in hemophilia patients with inhibitors. A phase 3 study for on-demand treatment of bleeding episodes (Crimson 1 study, NCT04489537) has been terminated.

### 4.2 New Bispecific Antibody

In addition to emicizumab, a new FVIIIa-mimetic bispecific antibody, Mim8 (denecimig), is currently under development (Figure 2). Preclinical studies have shown that Mim8 is more potent than emicizumab because it produces higher thrombin levels in plasma from patients with hemophilia A in vitro and leads to a greater reduction in bleeding frequency in mice with hemophilia A.^50^ Mim8 exhibits 97% bioavailability following subcutaneous injection in cynomolgus monkeys.^50^ In humans, the plasma half-life of Mim8 is approximately 25–35 days after a single subcutaneous injection, which could allow for weekly to monthly dosing.^51^

A phase 2 clinical study in a small cohort of patients with hemophilia A (FRONTIER 1) demonstrated that weekly and monthly (higher dose) subcutaneous injections of Mim8 resulted in 70%–90% of patients achieving zero bleeds, with a favorable safety profile.^42^ Ongoing phase 3 studies for prophylaxis, including FRONTIER 2, FRONTIER 3, and FRONTIER 4 (an open-label extension of the FRONTIER 1–3 trials), aim to further evaluate Mim8 in larger patient populations.^42,52^ FRONTIER 5 is another planned phase 3 trial designed to investigate the pharmacokinetics, pharmacodynamics, and safety of switching from emicizumab to Mim8 in patients with hemophilia A. Like emicizumab, Mim8 may interfere with certain laboratory tests, including the activated partial thromboplastin time and chromogenic assays containing human proteins.^53^

### 4.3 Antitissue Factor Pathway Inhibitor Antibody

Endogenous tissue factor pathway inhibitor (TFPI) is primarily synthesized by endothelial cells.^54^ It plays a role in inactivating the tissue factor/FVIIa complex.^28,54^ Therefore, targeting TFPI can enhance the tissue factor (extrinsic) pathway of coagulation, which might benefit patients with hemophilia. Concizumab (mAb 2021) is the first monoclonal antibody developed against TFPI (Figure 2). In a rabbit model of hemophilia, both intravenous and subcutaneous injections of mAb 2021 reduced bleeding following injury.^55^ A pharmacokinetic study in humans (including healthy volunteers and patients with hemophilia) showed that the half-life of concizumab is 30–74 h after intravenous injection or 75–115 h after subcutaneous injection.^56^

Results from phase 2 clinical studies in patients with hemophilia A (explorer4 trial) and in those with hemophilia A or B with inhibitors (explorer5 trial) showed that daily subcutaneous injections of 0.15 mg/kg concizumab, with the possibility of dose escalation to 0.20 and 0.25 mg/kg if three or more spontaneous bleeding episodes occurred within 12 weeks, significantly reduced bleeding frequency without severe adverse events over a 24-week period.^57^ In addition, the reduced bleeding rate was confirmed during a 2-year follow-up in the extension study.^58^ Injection site reactions were common side effects. A recent phase 3 study (explorer7 trial) of concizumab in patients with hemophilia with inhibitors initially used a loading dose of 1.0 mg/kg on day 1, followed by 0.25 mg/kg daily. However, the trial was paused because of thrombotic events, and patients were switched to on-demand treatment. The dosage regimen was then adjusted to a loading dose of 1.0 mg/kg followed by 0.2 mg/kg daily, with further dose adjustments based on plasma concizumab concentrations at week 4. If the plasma concentration was <200 ng/mL, the dose was increased to 0.25 mg/kg, and if it was >4000 ng/mL, the dose was decreased to 0.15 mg/kg.^43^ The results showed that prophylaxis with concizumab reduced bleeding episodes in these patients with a low rate of side effects (approximately 20% injection site reactions).^43^ Concizumab has been approved in Canada for prophylaxis in patients with hemophilia B and inhibitors, and it is under consideration in the United States, Europe, and Japan.^59^ It is recommended that patients discontinue concizumab before major surgery and resume it at the same maintenance dose within 10–14 days after surgery.^59^ Phase 3 trials of concizumab in patients with hemophilia without inhibitors, including explorer8 and explorer10, are ongoing.^60^

Marstacimab is a newly developed anti-TFPI monoclonal antibody (Figure 2). Following a 300-mg subcutaneous injection, the plasma half-life of marstacimab is approximately 65.8 h in healthy volunteers^61^ and 90.5 h in patients with severe hemophilia A or B with or without inhibitors,^62^ supporting the possibility of weekly administration. Data from a phase 1*b*/2 clinical study in patients with hemophilia A or B with or without inhibitors demonstrated that subcutaneous injection of 150 mg, 300 mg, or 450 mg or a 300-mg loading dose followed by 150 mg marstacimab once a week for 2 months significantly reduced the bleeding rate.^44^ Similar efficacy was observed during extended use for 1 year, without serious side effects or thrombosis.^63^ Results from the phase 3 BASIS trial in patients with severe hemophilia A or moderately severe to severe hemophilia B showed that a single subcutaneous loading dose of 300 mg marstacimab, followed by a weekly 150-mg dose, significantly reduced the bleeding rate over 12 months compared with previous on-demand and routine prophylaxis therapy.^45^ There were no reported thrombotic events during treatment with marstacimab.^45^ In October 2024, marstacimab was approved in the United States for prophylaxis in patients with hemophilia A or B without inhibitors. Notably, befovacimab, another anti-TFPI antibody in development, was terminated in a phase 2 clinical study because of thrombotic events.^64^

### 4.4 Small Interfering RNA against Antithrombin (AT)

AT is an important enzyme that inactivates thrombin, FXa, and to a lesser extent, FIXa, FXIa, and FXIIa.^65^ The activity of AT is further stimulated by heparan sulfate (an endogenous anticoagulant) and heparin derivatives.^65^ Therefore, targeting AT can enhance the activity of thrombin and FXa, potentially promoting hemostasis in patients with hemophilia.^66^ Fitusiran is the first small interfering RNA (siRNA) that specifically targets AT mRNA (mRNA) and induces its degradation primarily within hepatocytes (Figure 2).^66,67^

A monthly subcutaneous injection of fitusiran at a dose of 50 mg or 80 mg administered for 3 months in patients with moderate or severe hemophilia A or B and inhibitors demonstrated that the plasma half-life of fitusiran is 3–5 h.^68^ Despite this relatively short half-life, fitusiran has a prolonged duration of action. A reduction in AT activity is observed by day 7, with maximum reduction occurring on day 28.^68^ Phase 3 studies in patients with severe hemophilia A or B without inhibitors (ATLAS-A/B trial)^46^ and with inhibitors (ATLAS-INH trial)^47^ showed that prophylaxis with a subcutaneous injection of fitusiran at 80 mg once a month significantly reduced the bleeding frequency during a 9-month observation period. Additionally, patients who switched from bypassing agents or clotting factor concentrates to fitusiran (ATLAS-PPX trial) experienced a reduction in bleeding episodes compared with those who remained on bypassing agents or clotting factor concentrates.^48^

Common side effects of fitusiran include elevated levels of alanine aminotransferase (23%–32% with a mean duration of approximately 41 days), injection site reactions (5%–10%), and upper respiratory tract infections (5%–10%). A few thrombotic events have also been reported, with two cases occurring in both the ATLAS-INH and ATLAS-PPX studies.^46−48^

## 5 Conclusions

Managing hemophilia with inhibitors remains challenging, because of the limited therapeutic options available. Although ITI is the standard of care for inhibitor eradication, it is often costly because of the need for frequent administration of clotting factor concentrates. Moreover, not all patients respond well to this approach, with a reported success rate of approximately 70%–80%. The use of bypassing agents, such as rFVIIa and aPCC, has proven to be beneficial for prophylaxis and the acute treatment of bleeding episodes in patients with hemophilia with inhibitors. However, the need for an intravenous injection or infusion of these agents may be inconvenient for patients. Emicizumab, an FVIIIa-mimetic bispecific antibody, offers improved patient compliance because of its subcutaneous route of administration. However, it is currently approved only for prophylaxis in patients with hemophilia A. Recent advances in the development of nonfactor agents with different mechanisms of action, such as anti-TFPI antibodies (concizumab and marstacimab) and AT siRNA (fitusiran), hold promise for providing additional effective treatments for patients with hemophilia with inhibitors. Additionally, the rFVIIa variant (marzeptacog alfa) and the bispecific antibody Mim8 show potential because of their enhanced activity. The introduction of subcutaneous injections with less frequent dosing schedules (e.g., weekly or monthly) for these new agents may significantly reduce hospitalization and treatment burdens for patients, ultimately improving their quality of life.

## References

[ref1] Bolton-Maggs P. H. B.; Pasi K. J. Haemophilias A and B. Lancet 2003, 361 (9371), 1801–1809. 10.1016/S0140-6736(03)13405-8. 12781551

[ref2] Peyvandi F.; Garagiola I.; Young G. The past and future of haemophilia: diagnosis, treatments, and its complications. Lancet 2016, 388 (10040), 187–197. 10.1016/S0140-6736(15)01123-X. 26897598

[ref3] Srivastava A.; Santagostino E.; Dougall A.; Kitchen S.; Sutherland M.; Pipe S. W.; Carcao M.; Mahlangu J.; Ragni M. V.; Windyga J.; Llinás A.; Goddard N. J.; Mohan R.; Poonnoose P. M.; Feldman B. M.; Lewis S. Z.; van den Berg H. M.; Pierce G. F. WFH Guidelines for the Management of Hemophilia, 3rd edition. Haemophilia 2020, 26 (S6), 1–158. 10.1111/hae.14046. 32744769

[ref4] Meeks S. L.; Batsuli G. Hemophilia and inhibitors: current treatment options and potential new therapeutic approaches. Hematology Am. Soc. Hematol Educ Program 2016, 2016 (1), 657–662. 10.1182/asheducation-2016.1.657. 27913543 PMC6142469

[ref5] Jardim L. L.; Chaves D. G.; Rezende S. M. Development of inhibitors in hemophilia A: An illustrated review. Res. Pract Thromb Haemost 2020, 4 (5), 752–760. 10.1002/rth2.12335. 32685884 PMC7354390

[ref6] Patel T.; Shah S.; Bhatnagar N.; Gajjar M.; Shah M.; Tripathi S. Prevalence of Inhibitors in Hemophilia Patients and its Clinical Implications”: A Study of 276 Patients in Western India. Global Journal of Transfusion Medicine 2019, 4 (2), 168 10.4103/GJTM.GJTM_35_19.

[ref7] Angchaisuksiri P.; Amurao-Abiera M.; Chou S. C.; Chewcharat P.; Chozie N. A.; Gomez R.; Leng T. S.; Lin P. C.; Mai N. T.; Muda Z.; Seth T.; Sosothikul D.; Siu-Ming Wong R. Haemophilia care in Asia: Learning from clinical practice in some Asian countries. Haemophilia 2024, 30 (3), 609–616. 10.1111/hae.14998. 38523289

[ref8] Lieuw K. Many factor VIII products available in the treatment of hemophilia A: an embarrassment of riches?. J. Blood Med. 2017, 8, 67–73. 10.2147/JBM.S103796. 28670147 PMC5479262

[ref9] Graf L. Extended Half-Life Factor VIII and Factor IX Preparations. Transfus Med. Hemother 2018, 45 (2), 86–91. 10.1159/000488060. 29765290 PMC5939656

[ref10] Mannucci P. M. Hemophilia therapy: the future has begun. Haematologica 2020, 105 (3), 545–553. 10.3324/haematol.2019.232132. 32060150 PMC7049365

[ref11] Dargaud Y.; Leuci A.; Ruiz A. R.; Lacroix-Desmazes S. Efanesoctocog alfa: the renaissance of Factor VIII replacement therapy. Haematologica 2020, 109 (8), 2436–2444. 10.3324/haematol.2023.284498. PMC11290510 38356459

[ref12] Bjorkman S. A commentary on the differences in pharmacokinetics between recombinant and plasma-derived factor IX and their implications for dosing. Haemophilia 2011, 17 (2), 179–184. 10.1111/j.1365-2516.2010.02431.x. 21299739

[ref13] Tardy B.; Lambert T.; Chamouni P.; Montmartin A.; Trossaert M.; Claeyssens S.; Berger C.; Ardillon L.; Gay V.; Delavenne X.; Harroche A.; Chelle P. Revised terminal half-life of nonacog alfa as derived from extended sampling data: A real-world study involving 64 haemophilia B patients on nonacog alfa regular prophylaxis. Haemophilia 2022, 28 (4), 542–547. 10.1111/hae.14560. 35420242

[ref14] Tieu P.; Chan A.; Matino D. Molecular Mechanisms of Inhibitor Development in Hemophilia. Mediterr J. Hematol Infect Dis. 2020, 12 (1), e2020001 10.4084/mjhid.2020.001. 31934311 PMC6951349

[ref15] Schep S. J.; Schutgens R. E. G.; Fischer K.; Boes M. L. Review of immune tolerance induction in hemophilia A. Blood Rev. 2018, 32 (4), 326–338. 10.1016/j.blre.2018.02.003. 29482894

[ref16] Hermans C.; Astermark J.; De Moerloose P. Exposure to factor VIII and prediction of inhibitor development: exposure days vs. danger days, or both?. J. Thromb Haemost 2012, 10 (10), 2194–2196. 10.1111/j.1538-7836.2012.04871.x. 22846047

[ref17] Mahlangu J.; Oldenburg J.; Callaghan M. U.; Shima M.; Santagostino E.; Moore M.; Recht M.; Garcia C.; Yang R.; Lehle M.; Macharia H.; Asikanius E.; Levy G. G.; Kruse-Jarres R. Bleeding events and safety outcomes in persons with haemophilia A with inhibitors: A prospective, multi-centre, non-interventional study. Haemophilia 2018, 24 (6), 921–929. 10.1111/hae.13612. 30295389

[ref18] Prezotti A. N. L.; Frade-Guanaes J. O.; Yamaguti-Hayakawa G. G.; Ozelo M. C. Immunogenicity of Current and New Therapies for Hemophilia A. Pharmaceuticals (Basel) 2022, 15 (8), 911 10.3390/ph15080911. 35893734 PMC9331070

[ref19] Blanchette V.S.; Key N.S.; Ljung L.R.; Manco-Johnson M.J.; van den Berg H.M.; Srivastava A. Hemostasis. Definitions in hemophilia: communication from the SSC of the ISTH. J. Thromb Haemost 2014, 12 (11), 1935–1939. 10.1111/jth.12672. 25059285

[ref20] Ljung R.; Auerswald G.; Benson G.; Dolan G.; Duffy A.; Hermans C.; Jimenez-Yuste V.; Lambert T.; Morfini M.; Zupancic-Salek S.; Santagostino E. Inhibitors in haemophilia A and B: Management of bleeds, inhibitor eradication and strategies for difficult-to-treat patients. Eur. J. Haematol 2019, 102 (2), 111–122. 10.1111/ejh.13193. 30411401 PMC6936224

[ref21] Sherman A.; Biswas M.; Herzog R. W. Tolerance induction in hemophilia: innovation and accomplishments. Curr. Opin Hematol. 2018, 25 (5), 365–372. 10.1097/MOH.0000000000000446. 29994897 PMC10546904

[ref22] Astermark J. Immune tolerance induction in patients with hemophilia A. Thromb Res. 2011, 127 (Suppl 1), S6–9. 10.1016/j.thromres.2010.10.006. 21056905

[ref23] Li Z.; Chen Z.; Liu G.; Cheng X.; Yao W.; Huang K.; Li G.; Zhen Y.; Wu X.; Cai S.; Poon M. C.; Wu R. Low-dose immune tolerance induction alone or with immunosuppressants according to prognostic risk factors in Chinese children with hemophilia A inhibitors. Res. Pract Thromb Haemost 2021, 5 (5), e12562 10.1002/rth2.12562. 34278191 PMC8279128

[ref24] Holstein K.; Le Quellec S.; Klamroth R.; Batorova A.; Holme P. A.; Jimenez-Yuste V.; Astermark J. Immune tolerance induction in the era of emicizumab - still the first choice for patients with haemophilia A and inhibitors?. Haemophilia 2022, 28 (2), 215–222. 10.1111/hae.14470. 34918839

[ref25] Di Michele D. M. Immune tolerance induction in haemophilia: evidence and the way forward. J. Thromb Haemost 2011, 9 (Suppl 1), 216–225. 10.1111/j.1538-7836.2011.04349.x. 21781258

[ref26] Yoneyama K.; Schmitt C.; Portron A.; Kiialainen A.; Kotani N.; Jaminion F.; Retout S.; Adamkewicz J. I. Clinical pharmacology of emicizumab for the treatment of hemophilia A. Expert Rev. Clin Pharmacol 2023, 16 (9), 775–790. 10.1080/17512433.2023.2243213. 37529848

[ref27] Alcedo Andrade P. E.; Manucci P. M.; Kessler C. M. Emicizumab: the hemophilia A game-changer. Haematologica 2020, 109 (5), 1334–1347. 10.3324/haematol.2022.282099. PMC11063855 37916312

[ref28] Wichaiyo S.; Parichatikanond W.; Visansirikul S.; Saengklub N.; Rattanavipanon W. Determination of the Potential Clinical Benefits of Small Molecule Factor XIa Inhibitors in Arterial Thrombosis. ACS Pharmacol Transl Sci. 2023, 6 (7), 970–981. 10.1021/acsptsci.3c00052. 37470020 PMC10353063

[ref29] Shima M. Current status and future prospects of activated recombinant coagulation factor VIIa, NovoSeven(R), in the treatment of haemophilia and rare bleeding disorders. Ann. Hematol 2024, 103, 2647 10.1007/s00277-023-05287-2. 37391649 PMC11283401

[ref30] Roberts H. R.; Monroe D. M.; White G. C. The use of recombinant factor VIIa in the treatment of bleeding disorders. Blood 2004, 104 (13), 3858–3864. 10.1182/blood-2004-06-2223. 15328151

[ref31] Konkle B. A.; Ebbesen L. S.; Erhardtsen E.; Bianco R. P.; Lissitchkov T.; Rusen L.; Serban M. A. Randomized, prospective clinical trial of recombinant factor VIIa for secondary prophylaxis in hemophilia patients with inhibitors. J. Thromb Haemost 2007, 5 (9), 1904–1913. 10.1111/j.1538-7836.2007.02663.x. 17723130

[ref32] Young G.; Escobar M. A.; Pipe S. W.; Cooper D. L. Safety and efficacy of recombinant activated coagulation factor VII in congenital hemophilia with inhibitors in the home treatment setting: A review of clinical studies and registries. Am. J. Hematol 2017, 92 (9), 940–945. 10.1002/ajh.24811. 28589615

[ref33] Kempton C. L.; Meeks S. L. Toward optimal therapy for inhibitors in hemophilia. Blood 2014, 124 (23), 3365–3372. 10.1182/blood-2014-05-577643. 25428222

[ref34] Ghadimi K.; Levy J. H.; Welsby I. J. Prothrombin Complex Concentrates for Bleeding in the Perioperative Setting. Anesth Analg 2016, 122 (5), 1287–1300. 10.1213/ANE.0000000000001188. 26983050 PMC4840070

[ref35] Tjonnfjord G. E.; Holme P. A. Factor eight inhibitor bypass activity (FEIBA) in the management of bleeds in hemophilia patients with high-titer inhibitors. Vasc Health Risk Manag 2007, 3 (4), 527–531. 17969383 PMC2291336

[ref36] Leissinger C.; Gringeri A.; Antmen B.; Berntorp E.; Biasoli C.; Carpenter S.; Cortesi P.; Jo H.; Kavakli K.; Lassila R.; Morfini M.; Negrier C.; Rocino A.; Schramm W.; Serban M.; Uscatescu M. V.; Windyga J.; Zulfikar B.; Mantovani L. Anti-inhibitor coagulant complex prophylaxis in hemophilia with inhibitors. N Engl J. Med. 2011, 365 (18), 1684–1692. 10.1056/NEJMoa1104435. 22047559

[ref37] Oldenburg J.; Mahlangu J. N.; Kim B.; Schmitt C.; Callaghan M. U.; Young G.; Santagostino E.; Kruse-Jarres R.; Negrier C.; Kessler C.; Valente N.; Asikanius E.; Levy G. G.; Windyga J.; Shima M. Emicizumab Prophylaxis in Hemophilia A with Inhibitors. N Engl J. Med. 2017, 377 (9), 809–818. 10.1056/NEJMoa1703068. 28691557

[ref38] Jenkins P. V.; Bowyer A.; Burgess C.; Gray E.; Kitchen S.; Murphy P.; Platton S.; Riddell A.; Chowdary P.; Lester W. Laboratory coagulation tests and emicizumab treatment A United Kingdom Haemophilia Centre Doctors’ Organisation guideline. Haemophilia 2020, 26 (1), 151–155. 10.1111/hae.13903. 31859415

[ref39] Meeks S. L.; Leissinger C. A. The evolution of factor VIIa in the treatment of bleeding in haemophilia with inhibitors. Haemophilia 2019, 25 (6), 911–918. 10.1111/hae.13845. 31489759 PMC6899648

[ref40] Shapiro A. D.; Mitchell I. S.; Nasr S. The future of bypassing agents for hemophilia with inhibitors in the era of novel agents. J. Thromb Haemost 2018, 16 (12), 2362–2374. 10.1111/jth.14296. 30264916

[ref41] Mahlangu J.; Levy H.; Kosinova M. V.; Khachatryan H.; Korczowski B.; Makhaldiani L.; Iosava G.; Lee M.; Del Greco F. Subcutaneous engineered factor VIIa marzeptacog alfa (activated) in hemophilia with inhibitors: Phase 2 trial of pharmacokinetics, pharmacodynamics, efficacy, and safety. Res. Pract Thromb Haemost 2021, 5 (6), e12576 10.1002/rth2.12576. 34430790 PMC8371347

[ref42] Lentz S. R.; Chowdary P.; Gil L.; Lopez-Jaime F. J.; Mahlangu J.; Matytsina I.; Nielsen A. L.; Windyga J. FRONTIER1: a partially randomized phase 2 study assessing the safety, pharmacokinetics, and pharmacodynamics of Mim8, a factor VIIIa mimetic. J. Thromb Haemost 2024, 22 (4), 990–1000. 10.1016/j.jtha.2023.12.016. 38142846

[ref43] Matsushita T.; Shapiro A.; Abraham A.; Angchaisuksiri P.; Castaman G.; Cepo K.; d’Oiron R.; Frei-Jones M.; Goh A. S.; Haaning J.; Hald Jacobsen S.; Mahlangu J.; Mathias M.; Nogami K.; Skovgaard Rasmussen J.; Stasyshyn O.; Tran H.; Vilchevska K.; Villarreal Martinez L.; Windyga J.; You C. W.; Zozulya N.; Zulfikar B.; Jimenez-Yuste V.; explorer I. Phase 3 Trial of Concizumab in Hemophilia with Inhibitors. N Engl J. Med. 2023, 389 (9), 783–794. 10.1056/NEJMoa2216455. 37646676

[ref44] Mahlangu J. N.; Lamas J. L.; Morales J. C.; Malan D. R.; Salek S. Z.; Wang M.; Boggio L. N.; Hegemann I.; Mital A.; Cardinal M.; Zhu T.; Sun P.; Arkin S. A phase 1b/2 clinical study of marstacimab, targeting human tissue factor pathway inhibitor, in haemophilia. Br. J. Hamaetol. 2023, 200 (2), 229–239. 10.1111/bjh.18420. PMC10286764 35999026

[ref45] Matino D.; Acharya S.; Palladino A.; Hwang E.; McDonald R.; Taylor C. T.; Teeter J. Efficacy and Safety of the Anti-Tissue Factor Pathway Inhibitor Marstacimab in Participants with Severe Hemophilia without Inhibitors: Results from the Phase 3 Basis Trial. Blood 2023, 142 (Supplement 1), 285–285. 10.1182/blood-2023-181263.

[ref46] Srivastava A.; Rangarajan S.; Kavakli K.; Klamroth R.; Kenet G.; Khoo L.; You C. W.; Xu W.; Malan N.; Frenzel L.; Bagot C. N.; Stasyshyn O.; Chang C. Y.; Poloskey S.; Qiu Z.; Andersson S.; Mei B.; Pipe S. W. Fitusiran prophylaxis in people with severe haemophilia A or haemophilia B without inhibitors (ATLAS-A/B): a multicentre, open-label, randomised, phase 3 trial. Lancet Haematol 2023, 10 (5), e322–e332. 10.1016/S2352-3026(23)00037-6. 37003278

[ref47] Young G.; Srivastava A.; Kavakli K.; Ross C.; Sathar J.; You C. W.; Tran H.; Sun J.; Wu R.; Poloskey S.; Qiu Z.; Kichou S.; Andersson S.; Mei B.; Rangarajan S. Efficacy and safety of fitusiran prophylaxis in people with haemophilia A or haemophilia B with inhibitors (ATLAS-INH): a multicentre, open-label, randomised phase 3 trial. Lancet 2023, 401 (10386), 1427–1437. 10.1016/S0140-6736(23)00284-2. 37003287

[ref48] Kenet G.; Nolan B.; Zulfikar B.; Antmen B.; Kampmann P.; Matsushita T.; You C. W.; Vilchevska K.; Bagot C. N.; Sharif A.; Peyvandi F.; Young G.; Negrier C.; Chi J.; Kittner B.; Sussebach C.; Shammas F.; Mei B.; Andersson S.; Kavakli K. Fitusiran prophylaxis in people with hemophilia A or B who switched from prior BPA/CFC prophylaxis: the ATLAS-PPX trial. Blood 2024, 143 (22), 2256–2269. 10.1182/blood.2023021864. 38452197 PMC11181353

[ref49] Gruppo R. A.; Malan D.; Kapocsi J.; Nemes L.; Hay C. R. M.; Boggio L.; Chowdary P.; Tagariello G.; von Drygalski A.; Hua F.; Scaramozza M.; Arkin S.; Marzeptacog alfa Study Group I.; et al. Phase 1, single-dose escalating study of marzeptacog alfa (activated), a recombinant factor VIIa variant, in patients with severe hemophilia. J. Thromb Haemost 2018, 16 (10), 1984–1993. 10.1111/jth.14247. 30151972

[ref50] Ostergaard H.; Lund J.; Greisen P. J.; Kjellev S.; Henriksen A.; Lorenzen N.; Johansson E.; Roder G.; Rasch M. G.; Johnsen L. B.; Egebjerg T.; Lund S.; Rahbek-Nielsen H.; Gandhi P. S.; Lamberth K.; Loftager M.; Andersen L. M.; Bonde A. C.; Stavenuiter F.; Madsen D. E.; Li X.; Holm T. L.; Ley C. D.; Thygesen P.; Zhu H.; Zhou R.; Thorn K.; Yang Z.; Hermit M. B.; Bjelke J. R.; Hansen B. G.; Hilden I. A factor VIIIa-mimetic bispecific antibody, Mim8, ameliorates bleeding upon severe vascular challenge in hemophilia A mice. Blood 2021, 138 (14), 1258–1268. 10.1182/blood.2020010331. 34077951 PMC8499050

[ref51] Persson P.; Amstrup A. B.; Coester H. V.; Matytsina I.; Bas S. Mim8, a novel factor VIIIa mimetic bispecific antibody, shows favorable safety and pharmacokinetics in healthy adults. Res. Pract Thromb Haemost 2023, 7 (6), 102181 10.1016/j.rpth.2023.102181. 37745159 PMC10514552

[ref52] Seremetis S. V.; Clausen W. H. O.; Matytsina I.; Nissen S. M.; Wåhlander K. Mim8 Clinical Development Program: An Overview of the Frontier Studies. Blood 2022, 140 (Supplement 1), 5630–5631. 10.1182/blood-2022-163554.

[ref53] Bowyer A. E.; Kitchen S.; Ezban M. The effect of a next generation factor VIII mimetic bispecific antibody (Mim8) on assays of factor VIII activity and thrombin generation. J. Thromb Haemost 2023, 21 (3), 480–487. 10.1016/j.jtha.2022.12.023. 36696203

[ref54] Crawley J. T.; Lane D. A. The haemostatic role of tissue factor pathway inhibitor. Arterioscler Thromb Vasc Biol. 2008, 28 (2), 233–242. 10.1161/ATVBAHA.107.141606. 17951326

[ref55] Hilden I.; Lauritzen B.; Sorensen B. B.; Clausen J. T.; Jespersgaard C.; Krogh B. O.; Bowler A. N.; Breinholt J.; Gruhler A.; Svensson L. A.; Petersen H. H.; Petersen L. C.; Balling K. W.; Hansen L.; Hermit M. B.; Egebjerg T.; Friederichsen B.; Ezban M.; Bjorn S. E. Hemostatic effect of a monoclonal antibody mAb 2021 blocking the interaction between FXa and TFPI in a rabbit hemophilia model. Blood 2012, 119 (24), 5871–5878. 10.1182/blood-2012-01-401620. 22563084

[ref56] Chowdary P.; Lethagen S.; Friedrich U.; Brand B.; Hay C.; Abdul Karim F.; Klamroth R.; Knoebl P.; Laffan M.; Mahlangu J.; Miesbach W.; Dalsgaard Nielsen J.; Martin-Salces M.; Angchaisuksiri P. Safety and pharmacokinetics of anti-TFPI antibody (concizumab) in healthy volunteers and patients with hemophilia: a randomized first human dose trial. J. Thromb Haemost 2015, 13 (5), 743–754. 10.1111/jth.12864. 25641556

[ref57] Shapiro A. D.; Angchaisuksiri P.; Astermark J.; Benson G.; Castaman G.; Chowdary P.; Eichler H.; Jimenez-Yuste V.; Kavakli K.; Matsushita T.; Poulsen L. H.; Wheeler A. P.; Young G.; Zupancic-Salek S.; Oldenburg J. Subcutaneous concizumab prophylaxis in hemophilia A and hemophilia A/B with inhibitors: phase 2 trial results. Blood 2019, 134 (22), 1973–1982. 10.1182/blood.2019001542. 31444162 PMC6895373

[ref58] Shapiro A. D.; Angchaisuksiri P.; Astermark J.; Benson G.; Castaman G.; Eichler H.; Jimenez-Yuste V.; Kavakli K.; Matsushita T.; Poulsen L. H.; Wheeler A. P.; Young G.; Zupancic-Salek S.; Oldenburg J.; Chowdary P. Long-term efficacy and safety of subcutaneous concizumab prophylaxis in hemophilia A and hemophilia A/B with inhibitors. Blood Adv. 2022, 6 (11), 3422–3432. 10.1182/bloodadvances.2021006403. 35290453 PMC9198939

[ref59] Keam S. J. Concizumab: First Approval. Drugs 2023, 83 (11), 1053–1059. 10.1007/s40265-023-01912-6. 37341887

[ref60] Crescioli S.; Kaplon H.; Chenoweth A.; Wang L.; Visweswaraiah J.; Reichert J. M. Antibodies to watch in 2024. MAbs 2024, 16 (1), 2297450 10.1080/19420862.2023.2297450. 38178784 PMC10773713

[ref61] Cardinal M.; Kantaridis C.; Zhu T.; Sun P.; Pittman D. D.; Murphy J. E.; Arkin S. A first-in-human study of the safety, tolerability, pharmacokinetics and pharmacodynamics of PF-06741086, an anti-tissue factor pathway inhibitor mAb, in healthy volunteers. J. Thromb Haemost 2018, 16 (9), 1722–1731. 10.1111/jth.14207. 29908043

[ref62] Sun X.; Liu W.; Luo B.; Ma D.; Kalluru H.; Zhou Y.; Li J.; Peng A.; Liu Y.; Tong X.; Sun L.; Teeter J.; Raje S.; Yang R. Safety, tolerability, pharmacokinetics and pharmacodynamics of a single dose of marstacimab in Chinese participants with severe haemophilia. Haemophilia 2023, 29 (4), 1155–1159. 10.1111/hae.14814. 37339017

[ref63] Mahlangu J.; Luis Lamas J.; Cristobal Morales J.; Malan D. R.; Teeter J.; Charnigo R. J.; Hwang E.; Arkin S. Long-term safety and efficacy of the anti-tissue factor pathway inhibitor marstacimab in participants with severe haemophilia: Phase II study results. Br. J. Hamaetol. 2023, 200 (2), 240–248. 10.1111/bjh.18495. PMC10092220 36220152

[ref64] Mancuso M. E.; Ingham S. J. M.; Kunze M. an anti-tissue factor pathway inhibitor antibody: Early termination of the multiple-dose, dose-escalating Phase 2 study due to thrombosis. Haemophilia 2022, 28 (5), 702–712. 10.1111/hae.14595. 35667016 PMC9545794

[ref65] Rezaie A. R.; Giri H. Anticoagulant and signaling functions of antithrombin. J. Thromb Haemost 2020, 18 (12), 3142–3153. 10.1111/jth.15052. 32780936 PMC7855051

[ref66] Young G.; Lenting P. J.; Croteau S. E.; Nolan B.; Srivastava A. Antithrombin-lowering in hemophilia: a closer look at fitusiran. Research and Practice in Thrombosis and Haemostasis 2023, 7 (4), 100179 10.1016/j.rpth.2023.100179. 37358958 PMC10285540

[ref67] Machin N.; Ragni M. V. An investigational RNAi therapeutic targeting antithrombin for the treatment of hemophilia A and B. J. Blood Med. 2018, 9, 135–140. 10.2147/JBM.S159297. 30174468 PMC6110283

[ref68] Pasi K. J.; Lissitchkov T.; Mamonov V.; Mant T.; Timofeeva M.; Bagot C.; Chowdary P.; Georgiev P.; Gercheva-Kyuchukova L.; Madigan K.; Van Nguyen H.; Yu Q.; Mei B.; Benson C. C.; Ragni M. V. Targeting of antithrombin in hemophilia A or B with investigational siRNA therapeutic fitusiran-Results of the phase 1 inhibitor cohort. J. Thromb Haemost 2021, 19 (6), 1436–1446. 10.1111/jth.15270. 33587824 PMC8251589

